# Leaking pseudoaneurysm of lower limb saphenous vein graft: a rare complication and its successful treatment by endovascular embolization

**DOI:** 10.1259/bjrcr.20150445

**Published:** 2016-07-25

**Authors:** Shaileshkumar Garge, Pooja Vyas, Krantikumar Rathod, Sunila Jaggi, Inder Talwar

**Affiliations:** Department of Radiology and Intervention, Bombay Hospital and Medical Research Centre, Mumbai, India

## Abstract

A rare complication after lower limb revascularization using a saphenous vein bypass graft in a crush injury patient where the saphenous vein graft was the sole supplying vessel to the leg is described; a pseudoaneurysm developed in the saphenous vein graft and caused active profuse bleeding through the surgical wound. The aetiology of this condition is uncertain but it could occur owing to slippage of ligature from one of the tributaries of the saphenous vein. The diagnosis was made by digital subtraction angiography. The pseudoaneurysm was successfully obliterated by glue embolization, which stopped the bleeding immediately, with preservation of distal flow, thereby salvaging the limb.

## Background

Pseudoaneurysms of coronary saphenous vein graft are unusual and difficult to treat.^1^ Similarly, there is a possibility of a pseudoaneurysm developing in a lower limb saphenous vein graft; however, they have seldom been reported in published literature. Because the presentation of these conditions is generally dramatic and the mortality associated with an acute rupture is high, immediate treatment, either surgical or endovascular, is warranted.

We report a case in which a pseudoaneurysm developed in the lower limb saphenous vein graft from the anterior tibial artery (ATA) to dorsalis pedis artery (DPA) and caused profuse life-threatening bleeding through the surgical wound. As an alternative to surgical management, we used emergency glue embolization to repair the pseudoaneurysm, a technique that we believe has not been used before in such a case.

## Case history

**Figure 1.**
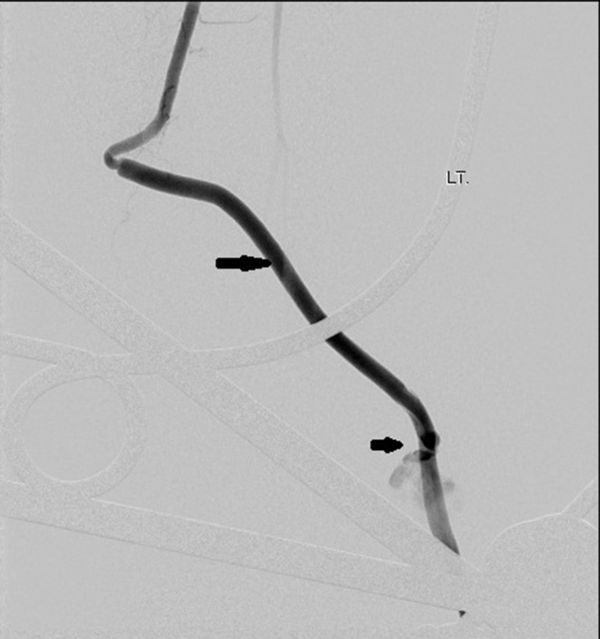
Digital subtraction angiography image of the left lower limb (anteroposterior view) showing the pseudoaneurysm (short arrow) arising from the middle of the saphenous vein (long arrow) graft without leak from the anastomotic site.

**Figure 2.**
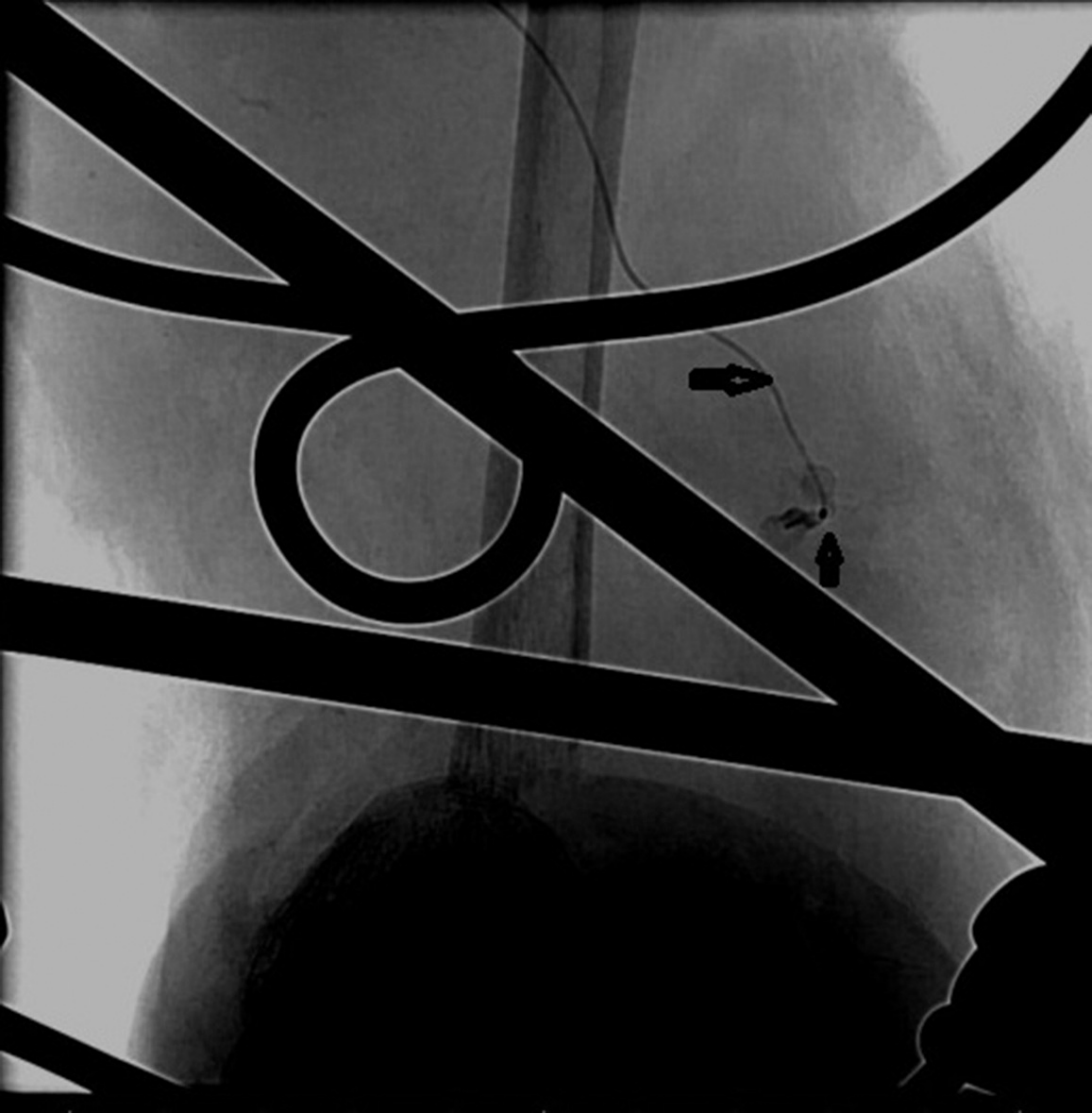
Digital substraction angiography image of the left lower limb (anteroposterior view) showing the tip of the microcatheter (long arrow) with injection of glue into the neck of the pseudoaneurysm (short arrow).

**Figure 3.**
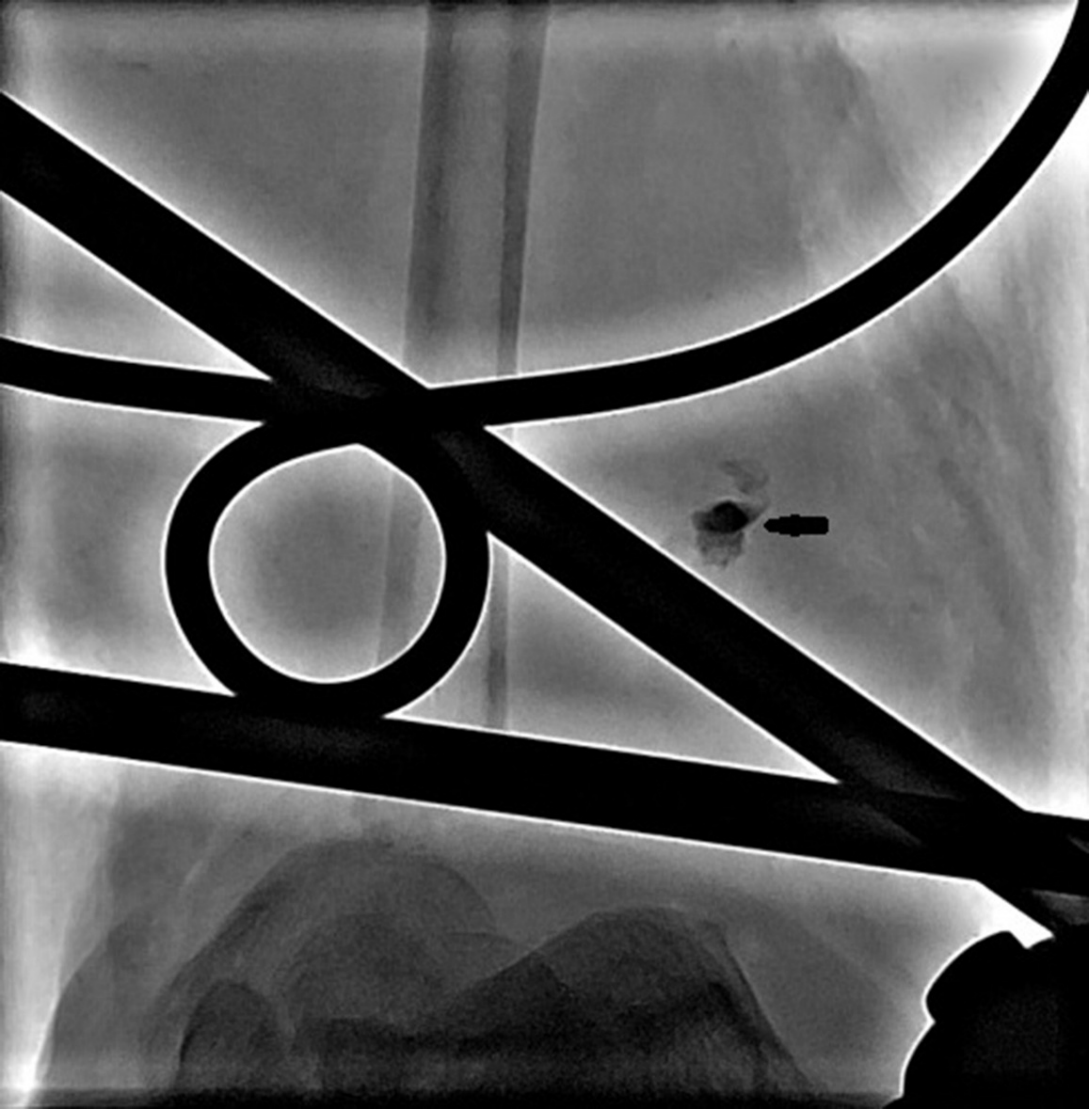
Digital subtraction angiography image of the left lower limb (anteroposterior view) showing formation of the complete glue cast (arrow).

**Figure 4.**
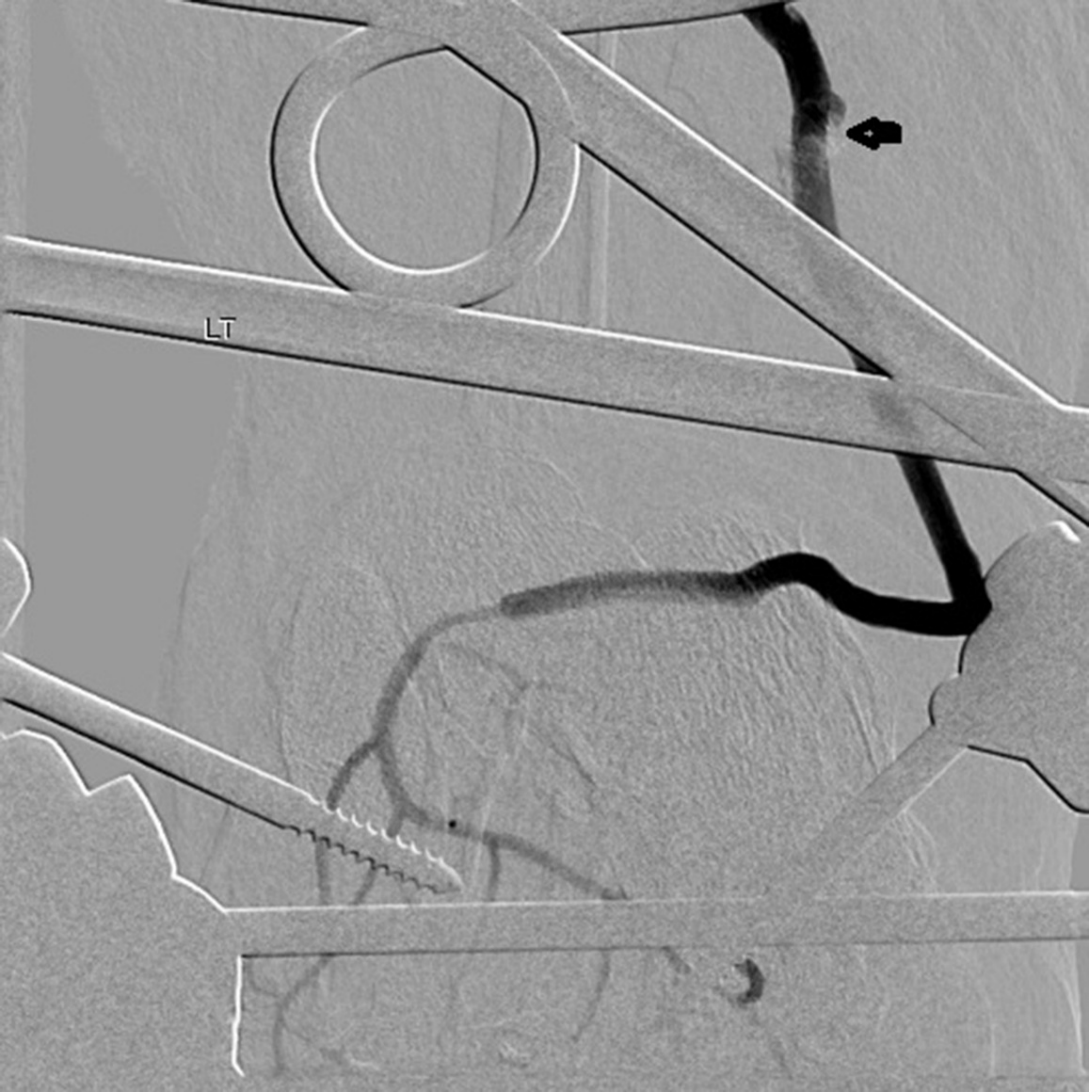
Digital subtraction angiography image of the left lower limb (anteroposterior view) showing complete exclusion of the pseudoaneurysm (arrow) from the circulation with good distal runoff in the dorsalis pedis.

A 34-year-old male presented with a history of crush injury to his left leg and multiple fractures involving the tibia and fibula. There was transection of the distal one-third of the ATA. He was treated with fracture fixation and saphenous vein graft from the ATA to the DPA. Immediately postoperatively, in the ward, the patient developed hypotension, with the foot turning pale and occurrence of bleeding from the operative site. An attempt was made to manage him conservatively with fluids, blood transfusion and local pressure, but his condition continued to worsen. Because of the suspicion of an anastomotic leak, digital subtraction angiography (DSA) was performed in the interventional radiology suite *via* the contralateral common femoral artery retrograde access using a 4 French sheath and 4 French Cobra catheter (Cook Medical, Bloomington, IN) which showed a pseudoaneurysm (Figure 1) that had developed owing to a leak from one of the tributaries of the saphenous vein that was used as graft; also there was no adequate flow from the posterior tibial artery and the peroneal artery (Figure 1). As the saphenous vein graft was the sole vessel supplying the foot, aggressive treatment of the pseudoaneurysm was considered. A microcatheter over a microwire (Progreat, Terumo, Tokyo, Japan) (Figure 2) was navigated through the Cobra catheter *via* the ATA, proximal artery venous graft anastomosis and the graft itself up to the neck of the pseudoaneurysm. The pseudoaneurysm was approximately 9 cm proximal from the distal anastomosis and measured 18 × 21 mm in size with a 3 mm neck. The tip of the microcatheter kept slipping out of the neck of the pseudoaneurysm into the parent vessel, so we abandoned coil embolization owing to the risk of non-target embolization. As glue was readily available with us, we injected 0.8 ml of glue [33% concentration *n*-butyl cyanoacrylate (NBCA) (Braun Medical Ltd, Sheffield, UK) by mixing 1 ml of glue with 2 ml of Lipiodol (Guerbet LLC, Bloomington, IN)] into the neck of the pseudoaneurysm after flushing the microcatheters with 5% dextrose solution. Soon a complete glue cast (Figure 3) formed inside the pseudoaneurysm and the microcatheter was pulled out by yanking. Post-embolization check DSA showed complete exclusion of the pseudoaneurysm from the circulation with good antegrade flow into the DPA (Figure 4). Local bleeding stopped immediately. Clinically, no further bleeding during the post-operative period was noted. Clinical follow-up after 4 weeks showed no local bleeding and good DPA pulsation.

## Discussion

Vein grafts are one of the most commonly used conduits for bypass grafting. The autologous saphenous vein is commonly used in vascular surgery as a bypass graft for relief of limb ischaemia and reconstruction of lower limb arteries such as the tibioperoneal and the DPA.^2–4^ Complications after surgical bypass include wound infection, necrosis, tissue loss, graft occlusion and bleeding.^5,6^

In our case, in view of the absence of any cause of bleeding, DSA was advised, which showed the pseudoaneurysm arising from the saphenous vein graft, probably owing to slippage of ligature from one of the tributaries. CT angiogram might help in identifying the source of bleeding, its location, extent and relationship with adjacent structures along with the extent of injury.

Traditionally, pseudoaneurysms have been managed surgically but with the advancement of endovascular intervention, optimal treatment is usually determined by the personal experience of the clinician. Treatment options include ultrasound-guided compression, percutaneous thrombin injection and endovascular stent graft placement. The success rate of thrombin injection (90–96%) is much higher than compression (75–78%). Stent graft placement can be considered as a bailout procedure.^7^

Angiography and embolization in vein grafts is extremely challenging, as veins have thin and fragile walls compared with arteries. The risk of vein graft perforation owing to percutaneous coronary intervention is estimated at 0.1%.^8^ Our case was complicated by the small size of the pseudoaneurysm neck, which was difficult to cannulate and get the microcatheter in a stable position. So owing to the risk of non-target embolization with use of pushable microcoils, we deferred coil embolization. We managed to keep the tip of the microcatheter just at the neck of the pseudoaneurysm and injected NBCA. NBCA is a permanent liquid embolic agent that turns radiopaque on mixing with Lipiodol or tantalum powder. It immediately polymerizes/solidifies on contact with an anion, such as hydroxyl moiety in water or various anions in blood.^9^

Intervention was thought to be successful when complete obliteration of the pseudoaneurysm sac was achieved and immediate stoppage of bleeding from the wound site was noted. Clinically, there was no further bleeding in the post-operative period. Clinical follow-up after 4 weeks also did not reveal any further bleeding and a good DPA pulsation was noted.

This case demonstrates the complexity of managing a saphenous vein graft pseudoaneurysm, in which it is difficult to place coils, with the help of glue embolization. This has seldom been reported in the literature.

## Learning points

1. Lower limb saphenous vein bypass graft pseudoaneurysm is a rare complication, the successful treatment of which with preservation of distal flow is critical, especially in patients with crush injury where the graft is the sole supplying vessel to the lower limb following surgery.
2. The small size of the pseudoaneurysm neck was difficult to cannulate and get the microcatheter in a stable position. Thus, owing to the risk of non-target embolization, glue embolization was preferred over pushable microcoils.
3. Awareness of ligature slippage as a common and important cause for pseudoaneurysm/bleeding when vascular grafts are used is required.

## Consent

Informed consent was obtained from the patient
